# Health financing for the poor produces promising short-term effects on utilization and out-of-pocket expenditure: evidence from Vietnam

**DOI:** 10.1186/1475-9276-8-20

**Published:** 2009-05-27

**Authors:** Henrik Axelson, Sarah Bales, Pham Duc Minh, Björn Ekman, Ulf-G Gerdtham

**Affiliations:** 1 Health Economics and Management, Institute of Economic Research, Lund University, Sweden; 2 Partnership for Maternal, Newborn and Child Health, c/o World Health Organization, 20 Avenue Appia,1211 Geneva 27, Switzerland; 3 Health Policy Unit, Ministry of Health, 138A Giang Vo St, Hanoi, Vietnam; 4 Department of Economics, Lund University, Sweden; 5 Division of Health Economics, Centre for Primary Health Care Research, Lund University, Sweden

## Abstract

**Background:**

Vietnam introduced the Health Care Fund for the Poor in 2002 to increase access to health care and reduce the financial burden of health expenditure faced by the poor and ethnic minorities. It is often argued that effects of financing reforms take a long time to materialize. This study evaluates the short-term impact of the program to determine if pro-poor financing programs can achieve immediate effects on health care utilization and out-of-pocket expenditure.

**Method:**

Considering that the program is a non-random policy initiative rolled out nationally, we apply propensity score matching with both single differences and double differences to data from the Vietnam Household Living Standards Surveys 2002 (pre-program data) and 2004 (first post-program data).

**Results:**

We find a small, positive impact on overall health care utilization. We find evidence of two substitution effects: from private to public providers and from primary to secondary and tertiary level care. Finally, we find a strong negative impact on out-of-pocket health expenditure.

**Conclusion:**

The results indicate that the Health Care Fund for the Poor is meeting its objectives of increasing utilization and reducing out-of-pocket expenditure for the program's target population, despite numerous administrative problems resulting in delayed and only partial implementation in most provinces. The main lessons for low and middle-income countries from Vietnam's early experiences with the Health Care Fund for the Poor are that it managed to achieve positive outcomes in a short time-period, the need to ensure adequate and sustained funding for targeted programs, including marginal administrative costs, develop effective targeting mechanisms and systems for informing beneficiaries and providers about the program, respond to the increased demand for health care generated by the program, address indirect costs of health care utilization, and establish and maintain routine and systematic monitoring and evaluation mechanisms.

## Introduction

Out-of-pocket (OOP) expenditure is the principal source of health financing in many developing countries, particularly in Asia [1]. In Vietnam, the share of OOP is around 64% of total health expenditure [2]. Given the low coverage of health insurance, specifically in the informal sector (which in Vietnam mainly consists of farmers and the self-employed), this exposes large parts of the population to two major risks. First, they may become impoverished or pushed further into poverty due to health expenditure [3]. Second, those households who delay or forego health care due to financial constraints are exposed to the detrimental impact of such decisions on their health status [4]. A study of Demographic and Health Surveys in 56 developing countries found that the poor consistently fared worse than their better-off peers in terms of health care utilization and health outcomes [5].

Different strategies have been used to try to improve access to health services by the poor and provide financial risk protection [6]. One strategy is to provide universal coverage of services to the whole population. The evidence on the effectiveness of this strategy is rather discouraging; public health expenditure often benefits the better-off more than the poor in developing countries, at least until universal coverage has been reached, which can take several years [7,8]. Subsidies may also benefit the rich after universal coverage has been fully achieved. Another strategy is to target interventions directly at the poor. Examples of such interventions include the *PROGRESA/Oportunidades *program in Mexico [9], health insurance for the poor in Colombia [10], and health insurance for the indigent in the Philippines [11].

The situation in Vietnam is very similar to that in other countries in Asia. OOP expenditure is the dominant source of health financing; almost 75% of total private expenditure is incurred at the point of use of health services [12]. This places a disproportionately heavier burden of health expenditure on the poor [13,14]. Wagstaff and van Doorslaer have estimated that OOP expenditures contributed to an increase in the number of absolute poor by 3.4 percentage points at the end of the 1990s [15]. Policies such as fee reductions, exemptions and free health insurance for the poor were initiated by the Government of Vietnam in the late 1990s, but coverage and effectiveness of these earlier policies were limited, partly due to insufficient central budget allocations to finance these policies [16]. For example, Nguyen found only limited or no impact of these programs on utilization and OOP expenditure [17]. It is therefore not surprising that inequalities in health utilization and outcomes widened between 1997 and 2002 [5]. For example, the ratio of under-five mortality for the poorest 20% vs. the richest 20% increased by 22%.

In 2002, in a major policy reform known as "Decision 139", the Government attempted to address the shortcomings of the previous policies by establishing the Health Care Fund for the Poor (HCFP) [18]. As with previous policies, the new strategy aimed at reducing financial barriers to health care access among vulnerable groups of the population, with the additional expectation that this would contribute to poverty reduction. In contrast to previous policies, however, Decision 139 committed the central government to back up the policy with significant financial resources [19]. Three-quarters of the total allocation per capita (USD 4.5 in 2002, more than a doubling of central government subsidies compared to earlier programs) is guaranteed by the central government; the rest is to be raised by the provinces. It also significantly increased the number of beneficiaries of government health financing policies; to 14.6 million people in 2002, which was equal to 18% of the total population. The target population is identified by methods developed to identify beneficiaries of the Hunger Eradication and Poverty Reduction program and includes a mix of individual characteristic and geographical targeting. Beneficiaries include households classified as poor according to the official poverty line, all ethnic minority residents of designated mountainous provinces, and all residents of communes classified as socio-economically disadvantaged. Provincial management boards were established to oversee program implementation. Decision 139 outlined two options for providing benefits; the management boards could purchase health insurance cards from the national health insurance system managed by Vietnam Social Security or directly reimburse health facilities for provision of services to the beneficiaries.

During the time period covered by this study, the program covered inpatient and outpatient care at public providers only. In 2005, revised health insurance regulations allowed for private sector provision of health insurance benefits, including to HCFP beneficiaries. However, to date very few private providers have been contracted to provide health insurance benefits. The package is generous, covering the costs of consultations, diagnosis, treatment, and rehabilitation during the time of treatment at the health facility; lab tests, diagnostic imaging, and other diagnostic techniques; medicines on the list drawn up by the MOH; blood and transfusions; medical procedures and surgery; use of materials, medical equipment, and treatment bed; and assistance at delivery. The package also includes some preventive interventions such as antenatal care. There are some exclusions to the benefits. For example, health insurance does not reimburse fees for certain health problems or treatments because of the elective nature of the intervention, or because those health problems are covered by other government programs, such as the Expanded Program for Immunization or disease-specific national programs. Benefits are valid directly upon issuance of the health insurance card. The Health Care Fund for the Poor covers costs up to 20,000,000 Vietnamese Dong (for each treatment episode), which was equivalent to USD 1,240 on January 1, 2004. The program also pays for transportation costs related to referral to higher-level facilities and some provinces have piloted payment of food costs for beneficiaries and caretakers.

Many recent studies have measured the effects of health insurance and other government-subsidized programs to increase access and financial risk protection of the poor in low and middle-income countries. Waters measured the impact of two health insurance programs in Ecuador and found mixed results: one of the programs had a strong positive association with use of curative care, but not for preventive care; the other program, directed at farmers, had positive but insignificant associations with both types of care [20]. Yip and Berman evaluated the impact of Egypt's school health insurance program and found that the program significantly improved access to health care, but increased inequalities as children not attending school tend to be poor and live in rural areas and thereby miss out on the program benefits [21]. Liu *et al. *studied the effects of urban health insurance reform in China and found that the reform led to significant increases in utilization of outpatient care by lower socioeconomic groups [22]. Hidayat *et al. *assessed the impact on equity of two mandatory health insurance schemes in Indonesia [23]. They found that the two schemes had a positive impact on access to care, but they did not find an impact on equity.

Trujillo *et al. *evaluated the impact of Colombia's subsidized health insurance program on health care utilization and found that the program significantly increased utilization among the country's poor and uninsured [24]. Wagstaff *et al. *evaluated the impact of China's New Cooperative Medical Scheme, which is a subsidized rural health insurance scheme, and found that the scheme increased utilization, but found no impact on OOP expenditure or on utilization among the poor [25]. Wagstaff and Yu estimated the impacts of a health reform project in China that combines supply-side interventions to improve quality of care with demand-side measures to expand health insurance and provide financial support to the very poor [26]. Their results suggest that the project reduced OOP and catastrophic expenditure and impoverishment due to health expenditure, but there was limited impact on utilization and mixed evidence regarding health outcomes.

Of particular relevance to the present study, Wagstaff examined the impact of Decision 139 in Vietnam using data from the 2004 Vietnam Household Living Standard Survey (VHLSS) [27]. Wagstaff used propensity score matching (PSM) with single differences to estimate the impact on health care utilization and expenditures, including differences in impact between the poorest groups compared to the rest of the eligible population. He found that while HCFP increased use of health services and reduced catastrophic expenditure, there was little impact on OOP expenditure and utilization in the poorest decile. The main contributions of this paper that add value vis-à-vis the paper by Wagstaff (2007) are as follows. First, we apply both single and double differences analysis, which enables us to estimate the impact when using panel data. Second, we use slightly different estimation techniques and it is important to analyze an issue in different ways, and with different methods, to establish validity of results. Finally, we have in-depth knowledge of the Vietnamese context, which enables us to interpret the findings in a policy relevant manner. The added value is further discussed in several parts of the paper.

The purpose of the present study is to evaluate the impact of the HCFP on health care utilization and expenditure in Vietnam during the initial period of implementation (2003–2004). The main contributions of our paper to the literature are three-fold. First, this paper constitutes the first systematic assessment using advanced non-randomized program evaluation techniques of what is the most ambitious health financing program in Vietnam to date and which is also of interest to other reforming countries. Second, we analyze the short-term impact of the program to determine if pro-poor financing programs can achieve immediate effects on key outcome indicators. Third, we draw policy lessons and recommendations for other low- and middle-income countries undertaking or considering similar health financing reforms.

In order to identify an appropriate methodology and choose relevant outcome variables, we formulated several research hypotheses. First, the HCFP would increase health care utilization. Second, utilization would be shifted from private to state facilities. Third, the use of self-treatment, such as purchasing of drugs directly from pharmacies and drug vendors, would decrease. Finally OOP and catastrophic health expenditures would decrease. To test our hypotheses, we compare the differences in utilization and expenditure outcomes between two groups of people who were eligible to receive benefits: those who had received a card and those who had not. (In a sensitivity analysis we conduct two additional comparisons.) We apply the technique of propensity score matching (PSM) with single differences in a cross-section analysis of VHLSS 2004, as well as a double differences analysis using a panel dataset from the VHLSS 2002 and 2004. In the study we find several important results.

The rest of this paper is organized as follows. The next section explains the data sources and methods. The following section presents the results. The final section concludes with a discussion of the results, policy implications and lessons learned for other low and middle-income countries currently undertaking similar health financing reforms.

## Methods

### Data sources

This study uses data from the 2002 and 2004 Vietnam Household Living Standard Surveys (VHLSS). Both datasets are relatively large and nationally representative and were collected through a stratified cluster sampling design. The 2004 survey contained a total of 9,188 households with 40,438 individuals. Almost one-third to half of the households interviewed in 2004 were previously interviewed in 2002. While the sample size in the panel data is thus smaller, the possibility of constructing a panel dataset for the impact evaluation was the main reason we chose the VHLSS as opposed to other datasets that may be richer in health variables, but that provide only cross-section data. The panel data allows us to eliminate bias from fixed effects. We will discuss the differences between the cross-section and panel datasets and the implications for the analysis in the Results and Discussion sections. Another important advantage of the VHLSS is that it contains a wide range of indicators that can be used to identify beneficiary status and determinants thereof. Such indicators are often missing in health-specific surveys. The VHLSS also contains sufficient data on health care utilization and expenditure. The 2002 survey serves as a baseline in comparisons of outcomes before and after implementation of the policy. The 2003–2004 period covered in the 2004 survey corresponds to the first year of implementation of the policy (Table 1). As discussed above, it should be noted that this allows for an assessment of the short-term impact; the full impact of the policy may not have emerged at this early stage. However, the program's scale and high cost, and the Government of Vietnam's expressed need to receive an early indication of its effectiveness in order to decide on the need for possible revisions to the policy, warrants an early study of the program's impact.

**Table 1:** Timeline of data sources and policy implementation

Year	2001				2002				2003				2004			
Quarter	1	2	3	4	1	2	3	4	1	2	3	4	1	2	3	4

Reference period of VHLSS 2002	x	x	x	x	x	x	x									

Decision 139 passed								x								

HCFP management boards set up									x	x	x	x	x	x	x	x

Reimbursement of health services begins											x	x	x	x	x	x

Reference period of VHLSS 2004										x	x	x	x	x	x	x

To estimate the impact on health care utilization, we examine the following population (outcome) indicators: number of inpatient admissions and outpatient visits to commune-level health facilities, district hospitals, and provincial or central hospitals in the public health system, and to any type of professionally trained private provider. In the 2002 VHLSS there were no separate variables for "district level hospitals" and "provincial/central hospital", so those variables could not be included in the panel analysis. Those variables could however be captured in the panel analysis as a group by the variable "all state hospitals". Inpatient admissions in private facilities were not included in the analysis due to their very low frequency in both 2002 and 2004.

To estimate the impact on health expenditure, we included the following variables: total household and per capita expenditure on health, household expenditure on inpatient and outpatient care and self-medication, and two measures of catastrophic expenditure: the proportion of households that incurred expenditure on health higher than 20% or 40% of non-food expenditure. We used household total expenditure as the measure of socio-economic status, given that wealth and consumption have been found to be unreliable indicators of socio-economic status. Table 2 reports descriptive statistics for the outcomes studied for both the full sample and those eligible for HCFP in 2004.

**Table 2:** Descriptive statistics – outcome variables, 2004

	Full sample		HCFP eligible	
Utilization	Mean	Standard error	Mean	Standard error

No. of outpatient (OP) visits in the 12-month recall period	0.990331	0.013564	0.784695	0.023113
no. of OP visits at commune level clinics	0.232479	0.005542	0.317338	0.011274
no. of OP visits at district hospital	0.119591	0.003903	0.110438	0.008076
no. of OP visits at provincial/central hospital	0.120778	0.004495	0.050821	0.005396
no. of OP visits at govt. hospital	0.240368	0.006001	0.161259	0.009783
no. of OP visits at private facility	0.493966	0.010409	0.289875	0.017243
No. of inpatient (IP) admissions in the 12-month recall period	0.095875	0.002429	0.107604	0.006065
no. of IP admissions at commune level clinics	0.014145	0.000728	0.022869	0.001745
no. of IP admissions at district hospital	0.03749	0.001434	0.047987	0.003491
no. of IP admissions at provincial/central hospital	0.040605	0.001564	0.031763	0.003538
no. of IP admissions at govt. hospital	0.078095	0.002242	0.07975	0.005576

Expenditure (in Vietnamese Dong, VND)				
Real per capita annual household health expenditure in the 12-month recall period	270.4715	3.8209	157.5965	5.038973
Real annual household health expenditure	1304.939	17.23415	798.0596	20.71008
Real annual household expenditure on OP care	463.8379	7.076816	262.0579	7.853138
Real annual household expenditure on IP care	522.9289	13.80825	333.0659	15.96045
Real annual household expenditure on self-medication	264.8263	2.639633	187.1645	4.547569
% of households with catastrophic health expenditure (20%)	0.184282	0.001928	0.210907	0.004033
% of households with catastrophic health expenditure (40%)	0.060537	0.001186	0.070563	0.002532

Source: Vietnam Household Living Standards Survey, 2004

Because provinces implemented the policy at different speeds, not all eligible beneficiaries had received a card by the time data for the VHLSS 2004 was collected. While this effect was obviously not positive for those who should have received cards in a more timely fashion, it was advantageous for the impact evaluation in that it allowed for comparison of impacts between people with very similar characteristics in a randomized way.

### Propensity score matching

We would like to observe the participants' outcomes with and without treatment. The treatment effect of an individual *i *can be written as:

(1)

where Y is the "outcome of interest"

However, it is clearly not possible to observe both outcomes for the same individual at the same time, which means that we cannot estimate the individual treatment effect τ_*i*_. Instead, we have to estimate population average treatment effects. The parameter that has received the most attention in the evaluation literature is the "average treatment effect on the treated" (ATT), which is the parameter that we will use in this study. ATT can be defined as:

(2)

where D is treatment status and takes a value of 1 for the treated and 0 for the untreated

To estimate impact of the HCFP, we apply the method of propensity score matching (PSM), which has been found to be a useful technique in the impact evaluation field [28]. In order to minimize selection bias associated with non-experimental data, the objective of matching is to find in a large group of non-participants those individuals who are similar to the participants in observed pre-treatment characteristics *X*. That being done, and assuming that the two groups do not differ in relevant unobservables, differences in outcomes between these two groups can be attributed to the program [29]. Rosenbaum and Rubin suggest the use of balancing scores *b*(*X*), i.e. functions of the relevant observed co-variates *X *that would be expected to be equal for both groups if the conditional distribution of *X *is independent of assignment into treatment [30]. One possible balancing score is the propensity score, i.e. the probability of participating in a program given observed characteristics *X*.

There is limited advice available regarding which functional form to use to estimate the propensity score (see e.g. the discussion in [31]). In principle any discrete choice model can be used. We estimated the propensity score with a logistic regression. The dependent variable in the regression indicates the treated and untreated (comparison) group. Because all eligible beneficiaries should be issued a health insurance card or a free health care card according to the implementation guidelines of the HCFP and a majority had been issued a card at the time of the 2004 VHLSS survey, we used possession of a card (or not) to indicate the treated and untreated (comparison) group in the cross-section analysis 2004. For the panel analysis 2002–2004, identification was based on possession of a card in the 2004 survey (as the 2002 data were collected before the policy was implemented), while explanatory variables were taken primarily from the 2002 survey. While two variables, illness and school attendance in the last 12 months recorded in the 2004 survey, may be affected by treatment (receipt of a card or not) and thus make them endogenous. While illness may affect whether or not someone receives a card because people with severe illness may receive greater sympathy from the people assessing poverty, it is unlikely that illness would be affected by whether someone receives a HCFP card or not. Utilization of health services, on the other hand, would be affected, as we hypothesize and which is supported by the results.

We do not think that receipt of a card would affect school attendance, as no money is given to the family when they have a card. However, if the family suffered an illness and had a card, they might be less likely to withdraw their children from school for lack of money. However, in our exploratory analysis of outcomes, we didn't find any effect on school enrollments when we included that as a potential outcome variable.

The objective in estimating the logistic regressions is to predict propensity scores that can be used to improve balance in the covariates between the treated and matched untreated groups, i.e. measured baseline covariates have a similar distribution in the two groups. Only covariates that could plausibly be expected to influence participation in the treatment, but that would not be expected to be influenced by the treatment, should be included in the model. Whether to include a large or small number of covariates is not resolved in the literature. The choice of variables should be based on economic theory and previous empirical findings. Following Rubin and Thomas [32], who argue that a variable should only be excluded from analysis if it is clear that the variable is either unrelated to the outcome or not a proper covariate, we included a relatively large number of covariates. We determined that, based on economic theory and our experience of the health sector in Vietnam and knowledge of the Health Care Fund for the Poor scheme, a number of covariates could plausibly influence participation in the treatment, but would not be expected to be influenced by the treatment. Among the covariates included were demographic variables, location of residence, income in 2002, poverty status, education and employment variables, illness in the past 12 months (2004), household size, and proportion of children and elderly in household (see Table 3 for a full list).

**Table 3:** Results of logistic regressions to estimate propensity score

Explanatory variables	Eligible with card vs. eligible without a card)	
	Cross-section 2002	Panel 2004
Female	-0.003	-0.023
	(-0.14)	(-0.68)

Age spline^1 ^(0–6)	**0.079**	0.023
	(2.58)**	(0.55)

Age spline (6–12)	**0.040**	0.040
	(2.47)*	(1.51)

Age spline (12–45)	0.002	**0.013**
	(0.48)	(2.12)*

Age spline (45+)	0.004	0.001
	(1.10)	(0.07)

Married	-0.155	**-0.381**
	(-1.81)	(-2.57)*

Registered in commune of residence	0.888	..
	(2.19)*	

Attended school during past 12 months (2004)	-0.215	-0.162
	(-2.64)**	(-1.26)

Ill during past 12 months (2004)	0.255	0.341
	(2.96)**	(2.56)*

Male head of household	0.021	0.458
	(0.11)	(1.47)

Head is ethnic minority in mountainous province	0.345	-0.029
	(1.38)	(-0.09)

Household classified as poor	**1.554**	0.355
	(9.86)**	(1.63)

Household has poor household certificate from commune		**0.839**
		(2.79)**

Head's occupation: skilled worker	-0.025	-0.129
	(-0.08)	(-0.26)

Head's occupation: unskilled worker	0.182	0.416
	(0.78)	(1.12)

Head's occupation: not working	0.331	0.223
	(1.20)	(0.48)

Individual employed in formal sector	**-0.647**	**-0.651**
	(-3.83)**	(-2.14)*

Head is ethnic Kinh or Chinese	**-0.536**	-0.328
	(-2.57)*	(-1.13)

Head completed primary schooling	-0.058	0.101
	(-0.45)	(0.52)

Head completed lower secondary schooling	0.037	0.076
	(0.22)	(0.33)

Head completed upper secondary schooling or above	0.212	**0.705**
	(0.90)	(1.97)*

Head's spouse in household	-0.046	-0.349
	(-0.23)	(-1.05)

Household size	**0.075**	0.010
	(2.86)**	(0.20)

Proportion of children <16 years in household	0.162	**0.988**
	(0.60)	(2.29)*

Proportion of adults >65 years in household	-0.089	0.548
	(-0.29)	(1.11)

Proportion of males in household	0.237	-0.100
	(0.86)	(-0.22)

Month of interview (2004)	**0.087**	0.065
	(2.13)*	(1.04)

Month of interview if reimbursement method changed between 2003 and 2004	**-0.079**	**-0.094**
	(-3.05)**	(-2.43)*

Urban location	-0.042	-0.001
	(-0.21)	(0.00)

135 commune	-0.079	**-0.704**
	(-0.45)	(-2.34)*

Commune 20+ kilometers from district capital	**0.389**	0.431
	(2.33)*	(1.64)

Remote commune	..	0.218
		(0.79)

Commune has pharmacy	..	-0.296
		(-1.28)

Northeast region	-0.150	-0.415
	(-0.48)	(-0.87)

Northwest region	0.360	-0.244
	(1.05)	(-0.46)

North Central region	-0.050	-0.414
	(-0.17)	(-0.87)

South Central region	0.378	0.435
	(0.92)	(0.75)

Central Highlands region	0.222	0.194
	(0.68)	(0.37)

Southeast region	-0.054	-0.231
	(-0.17)	(-0.42)

Mekong Delta region	-0.478	-0.527
	(-1.75)	(-1.12)

Real per capita monthly expenditures 2002	..	-0.001
		(-0.72)

Constant	**-3.549**	-1.258
	(-5.19)**	(-1.02)

Robust z statistics in parentheses

* significant at 5%; ** significant at 1%

Note: Statistically significant results (95% confidence level) are in boldface.

^1^Linear splines allow estimation of relationships between dependent and independent variables as a function composed of linear segments. Rather than restricting the coefficient to one value for the whole range of ages, it allows different coefficients for different ranges of ages. In our case, the spline is made up of 4 segments, the first from 0 to 6 years, the second from 7 to 12 years, the third from 13 to 45 years and the fourth from 45 years and higher. Within each variable, the years below the lowest level are set to 0, the years within the age range are set to the difference between the actual age and the highest age of the previous age range, and the ages above the upper age are set to the value for the highest age in the range.

The included covariates and the results of the logistic regressions that we used to construct the treated group and the matched, untreated group are presented in Table 3. The results are generally consistent with factors one would expect to be correlated with participation in the program. Greater likelihood of having a card was found in indicators such as being classified as poor according to the official poverty line, living in remote communes, and being interviewed later on in the year when provinces had more fully implemented the policy. Factors associated with lower likelihood of participation in the program included belonging to the majority ethnic group, employment in the formal sector, and higher educational level.

We checked on the range of the estimated propensity scores for both treated and untreated sub-samples to identify the region of common support. This is done to avoid biased estimates of impact that might result by matching treated and untreated cases that are very different from each other. Observations not under common support were dropped from the analysis.

We also investigated whether trimming of the sample was needed. If a large share of treated cases are concentrated in a small range of propensity scores while very few untreated cases fall into that same range then a given comparison may have to be used multiple times, and the estimated program impact will not be reliable in this range. It was determined that trimming was not needed. Table 4 presents the final sample sizes used to estimate program impact.

**Table 4:** Final sample size for impact analysis

	Have card	No card	Total
Single differences (Cross-section 2004)			
Overall sample (2004)	4,844	5,388	10,232
Sample with propensity score estimates	4,843	5,386	10,229
Sample under common support	4,843	5,375	10,218
Sample after trimming and in common support	4,843	5,375	10,218
Sample after matching	4,843	4,656	9,499

Differences in differences (Panel 2002–2004)			
Overall sample (2002–2004 Panel)	1,877	2,235	4,112
Sample with propensity score estimates	1,807	2,159	3,966
Sample under common support	1,784	2,151	3,935
Sample after trimming and in common support	1,784	2,151	3,935
Sample after matching	1,784	2,151	3,935

Nearest neighbor matching selects untreated cases with the closest propensity score to each treated case. Nearest n neighbors selects a group of n untreated cases with estimated propensity scores nearest to those of the treated group. This type of matching has a strong advantage in that the calculations are simple and the program runs quickly when the sample size is large. In this paper nearest neighbor matching was used with all the cross-sectional analysis. The number of nearest neighbors was selected based on the number that gave the best balance in covariates after matching (as discussed below). Thus in the cross-section, for the main comparison we used 6 nearest neighbors, and for the two comparisons in the sensitivity analysis we used 5 and 2 neighbors, respectively. In the panel, the best balance was achieved with kernel matching (as discussed below). However, the two sensitivity comparisons, matching with 11 and 2 nearest neighbors, respectively, resulted in a balance almost equivalent to that obtained with kernel matching, but with much shorter running time and with almost no difference in the impact estimates. Accordingly, we used n nearest neighbor matching to obtain the estimates for the two panel comparisons in the sensitivity analysis.

There are different kinds of matching algorithms that can be applied in PSM to match cases [29]. Based on preliminary tests, we used the nearest neighbor algorithm for the cross-section data, which selects a group of untreated cases with estimated propensity scores nearest to those of the treated group. The number of nearest neighbors used (2, 5, 6, or 11) varied with the sample, depending on which number achieved the best balance between the two groups. For the panel data we used kernel matching (with a bandwidth of 0.06).

The balance of distribution of observed characteristics between the treated group and the comparison group after matching is an important criterion for determining if matching was successful. Initial testing for covariate balance was done for the estimated propensity score using an algorithm developed by Dehejia and Wahba [33]. In this study, adding provincial and regional dummies to the equation led to important improvements in balance as the differential implementation of the policy across provinces plays an important role in determining whether or not a household has received a free health care card or health insurance for the poor.

We then analyzed two other indicators to further assess whether balance was achieved: a reduction of the pseudo R^2 ^of the logistic regression after matching and reduction in standardized bias as measured by the change in its mean, median and maximum. A low pseudo R^2 ^indicates that observable characteristics explain very little of the variation in the propensity scores in the treated and matched comparison sample. Matching led to substantial reductions in both indicators (Table 5). However, the results also indicate that the pseudo R^2 ^does not decrease to zero after matching, which means that matching reduces but does not entirely eliminate the potential bias in estimates of impact due to differences in observed covariates between the treatment and comparison cases.

**Table 5:** Pseudo R^2 ^and absolute standardized bias before and after matching

	Unmatched	Matched
Cross-section		
Pseudo R^2^	0.133	0.006
Mean bias	12.82	2.30
Median Bias	9.36	1.69
SD of bias	11.83	1.83
Minimum bias	0.14	0.06
Maximum bias	53.29	6.77
No. of Explanatory variables	38	38

Panel		
Pseudo R^2^	0.104	0.003
Mean bias	12.42	1.79
Median Bias	10.64	1.41
SD of bias	9.39	1.36
Minimum bias	0.24	0.01
Maximum bias	33.25	5.10
No. of Explanatory variables	42	42

### Estimating program impact

After suitable comparison groups had been formed using PSM, the next step in the analysis was to estimate the impact of Decision 139 on the selected utilization and expenditure indicators. This study obtained both single difference and double differences (or differences-in-differences) estimates. Single difference analysis compares the outcomes of the treated group to outcomes of the untreated group at a single point in time and was performed using cross-section data for 2004. Rosenbaum and Rubin showed that PSM is sufficient to remove bias due to all observed characteristics in large samples [30]. However, PSM does not reduce bias due to unobservable characteristics, which means that if unobservable characteristics determining selection into the treated group are correlated with outcomes, single difference estimates will be biased.

To address potential bias from unobservable characteristics, we also obtain double differences estimates to the panel dataset 2002–2004. First, we calculated the mean differences in outcomes before and after the intervention for the treated and untreated groups separately. Then we estimated program impact by calculating the difference between the mean differences of the two groups. The additional comparison across time eliminates bias resulting from time-invariant unobserved factors (i.e. fixed effects) that may be correlated with outcomes. Such "fixed effects" may include effects from variables such as unobserved community characteristics (for example, the unobserved quality of the available health services) or unobserved household/individual characteristics (for example, genetic endowments affecting health status). Bias from unobserved time-variant factors (for example, unobserved changes in the quality of available health services between 2002 and 2004) may still be present, but is assumed to introduce negligible bias.

Because the double differences analysis eliminates bias from fixed effects, the panel analysis may be considered preferable to the cross-section analysis. However, the VHLSS panel data may provide a downward biased estimate of the full impact of the Health Care Fund for the Poor because the 2002 baseline data incorporate the effects of previous government health care programs for the poor (which covered about 11% of the poor). We therefore present and discuss the results of both the cross-section and panel analyses.

We calculated standard errors for all impact estimates using bootstrapping techniques of 100 repetitions. There is some debate about whether bootstrapped standard errors are appropriate for use with certain matching methods, such as matching on the basis of one nearest neighbor [34], but it has not been established that it is inappropriate for kernel matching or for nearest neighbor matching using more than one nearest neighbor.

All calculations were computed using Stata^® ^version 9.2, including ado-files developed by Becker and Ichino [35] and Leuven and Sianesi [36].

## Results

We first report results of the cross-section analysis. Then, we present the findings of the panel data analysis. Finally, we report the results of a sensitivity analysis.

### Cross-section analysis with single differences

#### Impact on utilization

The results of the analysis of impact of the HCFP on utilization are presented in Table 6. The cross-section results indicate that the average number of outpatient visits and inpatient admissions was 4% and 6% higher, respectively, for the treated compared to the untreated. Neither of these differences is statistically significant. However, the small changes in overall utilization mask an interesting, and statistically significant, substitution effect: the treated group shifted from private to public providers. The results also indicate a substitution effect from primary to secondary and tertiary levels of care. For example, for outpatient care, the proportional impact was larger at the district and higher levels of outpatient care compared to commune-level facilities.

**Table 6:** Estimated impact on health care utilization

	Cross-section 2004						Panel 2002–2004
Variable	Average for treated	Average for untreated	Single difference in averages	% difference	Boot-strapped Std. Err.	P-value	Double differences/differences-in-differences (absolute)
No. of outpatient (OP) visits in the 12-month recall period	0.818	0.786	0.033	4%	0.060	0.585	0.06
no. of OP visits at commune level clinics	**0.380**	**0.308**	**0.072**	**23%**	**0.034**	**0.034**	**0.10**
no. of OP visits at district hospital	**0.144**	**0.078**	**0.066**	**85%**	**0.022**	**0.003**	Not available
no. of OP visits at provincial/central hospital	**0.051**	**0.029**	**0.022**	**74%**	**0.009**	**0.018**	Not available
no. of OP visits at govt. hospital	**0.195**	**0.107**	**0.088**	**82%**	**0.025**	**0.000**	0.08
no. of OP visits at private facility	**0.222**	**0.357**	**-0.135**	**-38%**	**0.039**	**0.000**	-0.12
No. of inpatient (IP) admissions in the 12-month recall period	0.125	0.118	0.007	6%	0.012	0.557	0.00
no. of IP admissions at commune level clinics	**0.026**	**0.037**	**-0.010**	**-28%**	**0.004**	**0.020**	0.00
no. of IP admissions at district hospital	**0.060**	**0.038**	**0.023**	**60%**	**0.008**	**0.003**	Not available
no. of IP admissions at provincial/central hospital	0.035	0.038	-0.002	-6%	0.006	0.721	Not available
no. of IP admissions at govt. hospital	0.096	0.075	0.021	27%	0.011	0.065	0.02

Note: Statistically significant results (95% confidence level) are in boldface.

We found a smaller and more mixed impact on the use of inpatient care (which is much less frequently reported than outpatient care). For example, the number of admissions to district hospitals and any state hospitals for inpatient care was higher for the treated compared to the untreated group, but the number of admissions to commune-level facilities and provincial and central level hospitals was higher among the untreated group.

#### Impact on out-of-pocket expenditure

We found a negative impact of the HCFP on six out of the seven expenditure variables in the cross-section analysis (Table 7): the treated group spent less than the untreated group on health care and were less exposed to catastrophic expenditure than non-beneficiaries at the lower threshold of 20%, but not at the higher 40% threshold. The impact was statistically significant for household expenditure (19% lower for the treated), household expenditure on inpatient care (27% lower), expenditure on self-medication (16% lower), and exposure to catastrophic health expenditure at the 20% threshold (18% lower).

**Table 7:** Estimated impact on health expenditure (in Vietnamese Dong, VND)

	Cross-section 2004						Panel 2002–2004
Variable	Average for treated	Average for untreated	Difference in averages	% difference	Boot-strapped Std. Err.	P-value	Double differences/differences-in-differences (absolute)
Real per capita annual household health expenditure in the 12-month recall period	122.061	142.286	-20.226	-14%	12.597	0.108	-2.95
Real annual household health expenditure	**615.193**	**757.514**	**-142.322**	**-19%**	**56.166**	**0.011**	-52.06
Real annual household expenditure on OP care	215.971	230.841	-14.870	-6%	25.490	0.560	55.07
Real annual household expenditure on IP care	**247.785**	**338.748**	**-90.963**	**-27%**	**37.684**	**0.016**	**-134.55**
Real annual household expenditure on self-medication	**144.558**	**171.236**	**-26.678**	**-16%**	**12.307**	**0.030**	19.37
% of households with catastrophic health expenditure (20%)	**0.210**	**0.258**	**-0.048**	**-18%**	**0.015**	**0.002**	-0.04
% of households with catastrophic health expenditure (40%)	0.074	0.072	0.002	3%	0.010	0.800	0.01

Note: Statistically significant results (95% confidence level) are in boldface.

### Panel analysis with double differences

#### Impact on utilization

The results of the panel analysis (last column of Table 6) were generally consistent with the findings of the cross-section analysis for the outpatient utilization variables and indicated a general, albeit small, increase in overall utilization and a shift from private to public care. However, the positive impact on the number of visits at the commune health center was the only statistically significant effect. For inpatient utilization, no or very small differences between the treated and the untreated group were found.

#### Impact on out-of-pocket expenditure

With the exceptions of expenditure on outpatient care and self-medication, the results of the panel analysis (last column of Table 7) displayed some differences compared to the findings of the cross-section analysis. The panel results indicated a larger reduction among the treated in terms of total household and per capita expenditure on health, inpatient care (the only variable with a statistically significant difference), and catastrophic expenditure at the 20% threshold. However, the opposite effect was found for expenditure on outpatient care and self-medication, which means that the ratio of outpatient vs. inpatient expenditure was lower in 2002, relative to the ratio in 2004. In other words, the residual impact, in 2004, of previous policies and programs to finance health care for the poor was higher for expenditure on outpatient compared to inpatient care. This lower effectiveness of previous programs in reducing spending on inpatient care compared to the HCFP is to be expected given the much lower levels of benefits or assistance for indirect costs in programs prior to HCFP.

### Sensitivity analysis

In addition to the comparison of impact for those eligible for benefits who had a card versus those who did not, which is where we expect to see the largest impact, we also explored two additional comparisons: between eligible beneficiaries with a card and those ineligible for the HCFP; and between eligible beneficiaries without a card and those ineligible for HCFP benefits. The reason for these additional comparisons is that the comparison between eligible with and without a card, assumes that those without a card did not receive any benefits from the policy. If this assumption holds, the comparison would measure the impact of the program on beneficiaries. However, it is possible that even those without a card may have received benefits, which seems to indeed have occurred. For example, some provinces reported that during the time before they could arrange to issue cards, the health care used by poor people would be reimbursed from the health care fund for the poor. This would lead to an underestimate of the program's impact, which is why we explored the two other comparisons. However, most eligible beneficiaries had been issued with a card at the time of the 2004 VHLSS survey. Furthermore, the first comparison is the main focus of the paper. The other comparisons are secondary and intended to identify any underestimate of impact because eligible without a card might also have received benefits.

Matching was more successful in the original comparison. The two measures of covariate balance – reduction in standardized bias and pseudo R^2 ^of the logistic regressions used to estimate the propensity scores – indicate that matching reduces bias significantly more in this comparison than in the additional two comparisons. The additional two comparisons also required a larger trimming of the sample, leaving fewer observations for analysis. The results for the first comparison can therefore be considered to be more reliable and are therefore reported in this paper. Nonetheless, the additional comparisons were generally, but not always, consistent with the findings of the first comparison.

We also explored outliers in the data. Outliers may result from interviewer recording errors or data entry errors, in which case they should be corrected or dropped. However, outliers may also be unusual, but correctly entered, observations, in which case they should be retained. The available information in the VHLSS did not allow us to distinguish between the two possibilities and hence to know whether the observations should be dropped or retained. Accordingly, and given that the outliers we identified seemed plausible and did not affect the results in a significant manner, all outliers were retained.

## Discussion and conclusion

### Discussion of methods

This study has evaluated the impact of the HCFP on utilization and expenditure during the initial period of the program. We applied propensity score matching to find a suitable comparison group for the treated population. We have presented the results of two alternative PSM-techniques to measure impact: single differences analysis of the 2004 cross-section and double differences analysis of the 2002–2004 panel.

One could make an argument for only presenting the results of the double differences analysis on the panel data, since it eliminates bias due to fixed effects. As such, double differences estimates are preferable to the single difference estimates. However, the impact of the HCFP cannot be measured in relation to a situation of no program using the panel data, since other programs to assist the poor and disadvantaged groups were in place prior to the HCFP. In other words, the baseline data of the VHLSS 2002 may reflect the impact of previous programs, such as health insurance for the poor (although these previous programs covered only about 11% of the poor with a reduced benefit package). This problem was raised by Wagstaff, who also suggested that the value of a double difference analysis would be further diminished because the panel 2002–2004 represents a minority of households and because the health utilization questions were different in the two surveys [27]. Apart from a much reduced sample size, which is important, the problem with the double differences analysis of the panel data is that it only provides an estimate of the marginal impact of HCFP over its predecessor programs.) Wagstaff therefore did not conduct an analysis of the panel data. Because of the potential problems with the double differences panel analysis, we have focused on the results of the single differences cross-section analysis.

However, it can be argued that confounding from previous programs is limited, given that they achieved such low coverage and limited impact on outcomes. Finally, the panel dataset contain more than 4,100 households, which should provide us with an adequate sample size for most outcomes (the main exception is inpatient care, which is much less frequently reported). Because of the important effect elimination of fixed effects can have on the results, and because we believe the double difference panel analysis is feasible, we have therefore presented the results of those as well. While the double differences results are less conclusive than the single differences, they do generally support the results of the single differences and point to the same general direction of the impact of the program, as we shall see in the next section. The combination of using both single differences and double differences to these data provides us with the ability to conduct a strong analysis of this program.

### Discussion of results

This study found that the program achieved positive outcomes in a short time-period. There was a small impact on overall utilization of health care. Given that there was a significant negative impact on OOP expenditure, the results also indicate a reduction in expenditure per health event or unit of utilization. The reduced OOP expenditure is consistent with a study by Sepehri *et al. *on the effects of health insurance in Vietnam [37]. Beneficiaries were less exposed to catastrophic health expenditure at the lower threshold of 20%. They also reduced their expenditure on self-treatment, which is consistent with findings of studies on the effect of insurance on self-treatment in Vietnam [38,39]. This result is particularly encouraging in a country like Vietnam, where purchases of prescription and non-prescription medicines from pharmacies and drug vendors with limited qualifications accounted for around 42% of all health expenditure between 2000 to 2003 [12].

The substitution effect away from private to public providers, and particularly to higher levels of care, may be an indication that the HCFP alleviated financial barriers to accessing higher levels of care and thus responded to an unmet need. Beneficiaries may have received better quality of care, since district and higher level hospitals are generally better equipped and staffed than commune level clinics and private practitioners. However, the implications of this response to the HCFP by its beneficiaries warrant careful consideration by policy-makers to inform planning and resource allocation decisions. Adequate financial and human resources must be put in place to meet the increased demand for services generated by the HCFP. Investments may be needed in upgrading community health centers and ensuring a functioning referral system to ensure that conditions are treated at the most appropriate and cost-effective level of care.

Moreover, given that the private sector still constitutes an important source of care for the poor and the ethnic minorities, it needs to be better integrated into the program. Quality of care could be strengthened by setting appropriate incentives through performance-based contracting, effective regulation, and training programs.

The study found a smaller impact on utilization of inpatient compared to outpatient care. A plausible explanation is that the program has not been able to fully address the financial barriers faced by the poor. Indirect costs of care, such as transportation, food, loss of income and the opportunity costs of the time of caregivers, can be substantial and tend to be larger for inpatient care compared to outpatient care.

The findings of recent studies that have analyzed the impact of the HCFP are generally consistent with this study's results. An analysis of MOH Health Information System data by Bales *et al. *found that utilization of the government health services increased more rapidly during the period 2003–2005 than during the period 1997–2002, and particularly in provinces in which HCFP beneficiaries are more than 20% of the population [19]. A small-scale evaluation in two provinces also found evidence of increased utilization and reduced health expenditure among beneficiaries of the program [40]. The findings of Wagstaff are less consistent with our results [27]. Wagstaff found that the HCFP substantially increased service utilization, especially inpatient care (while we found a larger increase in outpatient care), and reduced the risk of catastrophic expenditure (our results were inconclusive on this issue), but did not find evidence of a reduction in average OOP expenditure (our study did). However, some similarities in the results were identified. Similarly to this study, Wagstaff found a substantial and significant shift from private outpatient care to public outpatient care.

### Data Limitations

Most questions are consistent between the 2002 and 2004 VHLSS surveys. However, changes were made to improve data collection in the health section between the 2002 and 2004 surveys, which led to some problems in comparability. While both surveys used a 12-month recall period, the VHLSS 2004 questionnaire on outpatient care included questions on illness to help respondents recall health care utilization, and allowed recording of multiple visits to the same facility on the same line. These changes presumably helped respondents to recall utilization events and might therefore have led to an upward bias in utilization figures for the VHLSS 2004 compared to the VHLSS 2002. However, we believe that it is unlikely that any reporting errors are systematically different between beneficiaries and non-beneficiaries of the program. Another limitation is that the VHLSS does not contain variables on timeliness of seeking care and length of stay. Finally, the VHLSS 2002 did not separate the level of government hospital.

### Lessons for low and middle-income countries

The design, implementation and outcomes of health reforms are often context-specific and may not always be transferable to other countries. However, there are lessons from Vietnam's early experiences with the HCFP that may be applicable to other low and middle-income countries that are planning or undergoing similar health reforms designed to increase health care access and utilization, and reduce the financial burden of health expenditures.

First, the results indicate that it is possible to achieve positive impact on outcomes such as health care utilization and out-of-pocket expenditure in a short-term period. This is critically important for policy makers, who are often under pressure to show quick results and to show that pro-poor financing reforms have their intended consequences.

Second, the failure of the programs in the 1990s suggest that it is critical that any efforts to target the poor are backed by adequate and sustained funding to ensure that providers are adequately reimbursed, particularly given their increased workload as a result of increased utilization due to the HCFP scheme. Inadequate reimbursement of providers was one of the reasons why the previous programs in Vietnam failed [41]. Vietnam has benefited from strong and sustained economic growth over the past 15 years, which has allowed the central government to provide most of the financing for the HCFP. Other countries will need to create the necessary fiscal space to ensure that pro-poor financing reforms are funded in a sustainable manner [42].

Third, the HCFP has benefited from effective targeting with limited leakage. The program took advantage of other targeting mechanisms and a decentralized and pragmatic approach to the identification of HCFP beneficiaries [43]. Other countries with limited resources can benefit from a similar approach.

Fourth, the HCFP is implemented through the national health insurance system, which has reduced transaction costs and also increased transparency through a uniform benefit package. However, the HCFP benefit package is quite generous and has increased the financial strain on the health insurance system. The scope of the benefit package and its implications for sustainability is something that other countries planning and implementing similar reforms need to carefully consider and monitor. Furthermore, VSS uses fee-for-service with budget ceilings as a reimbursement method and this provides perverse incentives to providers. This has been particularly problematic in the voluntary health insurance scheme, where provider-induced demand, moral hazard and adverse selection have led to significant over-spending. If the over-spending is not contained, in the long-run this will present a serious threat to the sustainability of the overall health insurance system, including the HCFP. In a sense this is unfair to the HCFP, since financial reports suggest that none of the provinces has over-spent its HCFP budget allocations.

Fifth, it is critical that provinces and central governments ensure that there is capacity to manage any financing program for the poor. In Vietnam, the delay in distributing cards for the HCFP, responding to queries of beneficiaries related to waiting time, and information about the use of the card could be attributed to a lack of capacity, which can adversely effect the implementation and finally the impact of HCFP policy. For example, lack of knowledge and information and the limited responsiveness of the system prevent some beneficiaries from seeking seek care under HCFP.

Sixth, addressing indirect costs of health care utilization is important. The HCFP includes some direct subsidies for transport and food. However, an argument could be made that because of high transaction costs such funds might be better spent on establishing a functioning referral system, including adequate transportation to higher levels of care.

Finally, it is important that systematic and routine monitoring and impact evaluation efforts are built into the design, implementation, and funding of health reform programs. For example, Mexico has invested significant efforts and resources in developing capacity to monitor and evaluate the impact of its health reform programs [44]. Vietnam, on the other hand, did not strengthen administrative capacity to manage the scheme, not did it explicitly plan for impact evaluations of the HCFP. This study took advantage of the availability of national household survey data, the nature of which allowed for measurement of the program's impact.

### Suggestions for future research

While the HCFP would benefit from routine collection of data for monitoring purposes, the analysis of future living standard surveys can complement such efforts and will be useful for exploring the medium and long-term impact of the program, including effects on living standard measures, such as income, consumption and poverty, and health outcomes, which take longer to emerge than effects on utilization and expenditure.

## Competing interests

The authors declare that they have no competing interests.

## Authors' contributions

HA performed statistical analysis and drafted the manuscript. SB developed the analytical approach and econometric methods. PM performed statistical analysis and contributed to the drafting of the manuscript. BE and UG contributed to the drafting of the manuscript. All authors read and approved the final manuscript.

## Author information

HA is a doctoral student at Lund University and a health economist at the Secretariat of the Partnership for Maternal, Newborn and Child Health in Geneva. In 2004–2007, he worked for the World Health Organization in Hanoi providing technical assistance on health insurance and the Health Care Fund for the Poor to the Ministry of Health and Vietnam Social Security.

SB is a health economist working at the Vietnamese Ministry of Health since 1999. She has worked on various empirical studies on health care for the poor and is currently focusing on hospital costing and payment reforms in Vietnam's health sector.

PM is a public health specialist at the Health Policy Unit and Health Care Support to the Poor of the Northern Uplands and Central Highlands of Vietnam (HEMA) project, Ministry of Health of Vietnam. He has worked on national health care for the poor policy and is currently focusing on providing technical assistance to provinces to improve quality of health services provided to the poor and ethnic minorities living in mountainous and disadvantaged regions.

BE is a health economist at Lund University. His research interests focus on health financing in low- and middle-income countries and on the economics of maternal and child health. He has worked as a health economics advisor to the Ministry of Health in Vietnam.

UG is a Professor in Economics at the Economics Department of University of Aberdeen. He is also Theme leader of the Program on Health Inequalities at the Health Economics Research Unit (HERU) at the University of Aberdeen. He is also the Principal Investigator of the Health Economics Program at Lund University.
